# Triterpenoids from *Gymnema sylvestre* and Their Pharmacological Activities †

**DOI:** 10.3390/molecules190810956

**Published:** 2014-07-28

**Authors:** Giovanni Di Fabio, Valeria Romanucci, Anna De Marco, Armando Zarrelli

**Affiliations:** 1 Department of Chemical Sciences, University Federico II, Complesso Universitario Monte S. Angelo, Via Cintia 4, IT-80126 Napoli, Italy; E-Mails: difabio@unina.it (G.D.F.); valeria.romanucci@unina.it (V.R.); 2 Department of Biology, University Federico II, Complesso Universitario Monte S. Angelo, Via Cintia 4, IT-80126 Napoli, Italy; E-Mail: anna.demarco@unina.it

**Keywords:** *Gymnema sylvestre*, triterpenoids, oleanes, pharmacological activities, phytochemistry

## Abstract

Because plants are estimated to produce over 200,000 metabolites, research into new natural substances that can be used in the pharmaceutical, agrochemical and agro-industrial production of drugs, biopesticides and food additives has grown in recent years. The global market for plant-derived drugs over the last decade has been estimated to be approximately 30.69 billion USD. A relevant specific example of a plant that is very interesting for its numerous pharmacological properties, which include antidiabetic, anticarcinogenic, and neuroprotective effects is *Gymnema sylvestre*, used as a medicinal plant in Asia for thousands of years. Its properties are attributed to triterpenoidic saponins. In light of the considerable interest generated in the chemistry and pharmacological properties of *G. sylvestre* triterpenes and their analogues, we have undertaken this review in an effort to summarise the available literature on these promising bioactive natural products. The review will detail studies on the isolation, chemistry and bioactivity of the triterpenoids, which are presented in the tables. In particular the triterpenoids oxidised at C-23; their isolation, distribution in different parts of the plant, and their NMR spectral data; their names and physico-chemical characterisation; and the biological properties associated with these compounds, with a focus on their potential chemotherapeutic applications.

## 1. Introduction: Medicinal Plants and Their Secondary Metabolites

Plants form an important part of our diet, and plant constituents and their nutritional value have been intensively studied for decades. In addition to primary metabolites (carbohydrates, lipids and amino acids), plants are able to synthesise a wide variety of compounds referred to as secondary metabolites. These are defined as compounds that do not have a well-recognised role in the essential processes of the plant but play an important role in the interactions of the plant with the environment [1]. Despite the term “secondary”, these compounds confer selective advantages to plant species by suppressing the growth of weeds; providing protection from predators, pathogens and abiotic stress; attracting pollinators, providing benefits with respect to animals and microorganisms; and serving as signals in interactions with other plants [2,3,4,5,6,7,8]. In addition, they play a role at the cellular level as growth regulators, modulators of gene expression and signal transducers [9]. Because plants are estimated to produce over 200,000 metabolites [10], research into new natural substances that can be used in the pharmaceutical, agrochemical and agro-industrial production of drugs, biopesticides, and food additives has grown in recent years. The yield of these compounds is often low (less than 1% dry weight) and depends greatly on the physiological and developmental stage of the plant [11]. Of the more than 400,000 species of plants, the phytochemicals of only a small percentage have been studied, and of these phytochemicals, only a small percentage has been examined for their biological properties because this research is complex and expensive. Two-thirds of medicinal plants are collected from the wild, but in Europe, only approximately 10% of plants used for commercial purposes are cultivated [12]. Currently one fourth of all prescribed pharmaceuticals in industrialised countries contains compounds that are directly or indirectly, via semi-synthesis, derived from plants. Furthermore, 11% of the 252 drugs considered as basic and essential by WHO are exclusively derived from flowering plants [13]. The global market for plant-derived drugs has been estimated to be approximately 30.69 billion USD annually over the last decade, and phytochemicals such as terpenes and steroids represent the most significant fraction with estimated annual sales of 12.4 billion USD [14].

Higher plants are rich source of bioactive constituents or phyto-pharmaceuticals used in the pharmaceutical industry. Many of these pharmaceuticals are still in use today, and often, useful synthetic substitutes have been found that possess the same efficacy and pharmacological specificity [15]. In addition to the field of pharmacology, secondary metabolites from plant extracts can also be used in the agrochemical sector. The recent interest in chemical to be used as defences for agricultural crops with less impact on the environment from operators in the sector and consumers has stimulated research into the isolation of new molecules of natural origin for use as biopesticides [9].

Over 60% of cancer drugs and 66% of antimicrobial compounds on the market today (including antibacterial, antifungal and antiviral compounds) are natural products or are derived from them [16]. Despite the difficulties associated with the study and isolation of phytochemicals, they have the advantage of greater efficacy than synthetic drugs for some diseases and a lower incidence of side effects. Additionally, a large percentage of the world’s population has no access to conventional pharmacological treatments. The different reasons mentioned above describe a situation in which research has ample space to improve and simplify extraction protocols and increase the levels of metabolites in plants of interest, through classical techniques of genetic improvement or innovative biotechnological approaches aimed at modifying biosynthetic pathways, in addition to testing substances already isolated for the widest possible spectrum of biological activities.

### 1.1. Terpenes and Terpenoids

Among the many biologically active substances of plant origin, a great deal of attention has been paid to terpenes, which are composed of isoprene subunits. More than 30,000 terpenes have been isolated thus far [17]. Terpenes are classified as mono-, sesqui-, di-, ses-, tri-, and tetraterpenes according to the number of isoprenoid units. The carbon skeleton may be acyclic, or they may possess mono-, bi-, tri-, tetra-, and pentacyclic structures [18,19,20,21,22,23,24]. Terpenes are of widespread and can be found in all organisms, both prokaryotic and eukaryotic. However, most bioactive terpenes have been detected in higher plants. Whereas mono- and sesquiterpenes are predominantly found in essential plant oils, the higher terpenes, including triterpenes, are found in balsams and resins [25,26]. Triterpenoids in their free form (sapogenins), bound to glycosides (saponins) or acetylated are particularly important and are ubiquitous throughout the plant kingdom; both *in vitro* and *in vivo* studies have shown that they also have important biological functions. Because of their relatively complex structures, terpenoids are usually referred to by trivial names instead of using systematic IUPAC nomenclature.

### 1.2. Classification of Terpenoids and Their Biological Effects

Based on the number of sugars present, saponins are classified as monodesmosides, with a single saccharide chain generally linked to C-3; bidesmosides, with two sugar chains linked to C-3 and C-28; and tridesmosides, with three saccharide chains. The sugars can be linear or branched; the most widespread saccharides in triterpenoid saponins are d-glucose, d-galactose, l-arabinose, l-rhamnose, d-xylose, d-fucose and glucuronic acid [27]. The biological effects of such terpenoids are very different, and can be summarised as follows: antitumor, antiviral, antidiabetic, anti-inflammatory, hepatoprotective, analgesic, antimicrobial, antimycotic, virostatic, immunomodulatory, and tonic activities [17]. A few compounds, such as corosolic acid, a dietary supplement against diabetes, are already on the market, and several others are in clinical trials or are to be launched soon. Many triterpenes exhibit significant biological activity, but several triterpenoids possess haemolytic and cytostatic properties that restrict their pharmaceutical use. To overcome these limitations and to expand the range of usable triterpenes, transformation of the compound by chemical or biotechnological techniques is possible. They are also of extreme interest to the agrochemical field because they are involved in the defence mechanisms of plants. Their biological activities are mainly related to interactions with cell membranes. The main mechanism of action of saponins against fungi appears to be related to their ability to form complexes with sterols in fungal membranes by altering the membrane integrity [28].

## 2. Triterpenoids from *Gymnema sylvestre* and Their Pharmacological Activities

The triterpenoidic saponins also act against insect pests by forming an insoluble complex with cholesterol, which is the precursor in the biosynthesis of the hormone ecdysone. Because cholesterol is the only source of sterols for the majority of the insects and a main energy supply, the cholesterol linked to saponins cannot be used, which leads to insect death [29].

A relevant specific example of a plant that is very interesting for its numerous pharmacological properties, which include antidiabetic, anticarcinogenic, and neuroprotective effects is *Gymnema sylvestre*, used as a medicinal plant in Asia for thousands of years. Its properties are attributed to triterpenoidic saponins. In light of the considerable interest generated in the chemistry and pharmacological properties of *G. sylvestre* triterpenes and their analogues, we have undertaken this review in an effort to summarise the available literature on these promising bioactive natural products.

**Figure 1:**
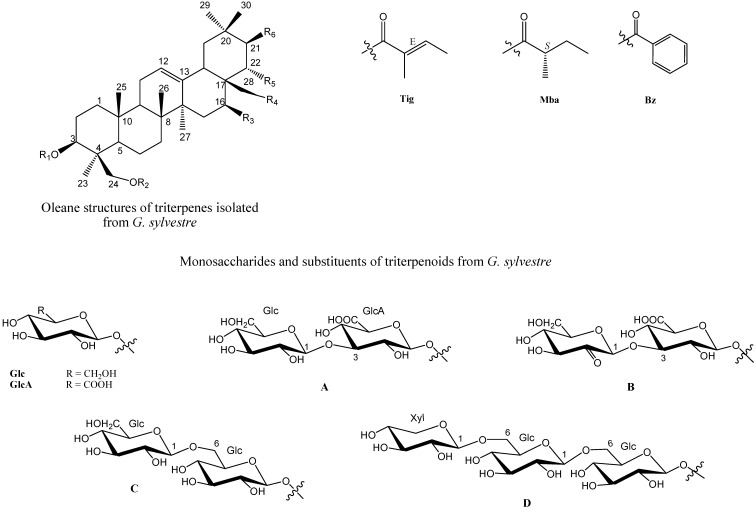
Chemical structures of triterpenes isolated from *G. sylvestre*.

The review will detail studies on the isolation, chemistry and bioactivity of the triterpenoids, which are presented in the following tables: triterpenoids oxidised at C-23 (Table 1 and Figure 1); their isolation, distribution in different parts of the plant, and their NMR spectral data (Table 2); their names and physico-chemical characterisation (Table 3 and Table 4, respectively); and, finally, the biological properties associated with these compounds, with a focus on their potential chemotherapeutic applications (Table 5).

### Biological Effects of Triterpenoids

Diabetes mellitus (DM) is a disease caused by a deficiency or diminished effectiveness of endogenous insulin. It is characterised by hyperglycaemia, deranged metabolism and sequelae predominantly affecting the vasculature. There are two main types of diabetes mellitus: Type 1, in which the body does not produce sufficient insulin; Type 2, due to the resistance to the insulin, often initially with normal or increased levels of circulating insulin. In the UK, there were 2.9 million people with diabetes in 2011. It is estimated that 5 million people will have diabetes in the UK by 2025. It is also estimated that there are approximately 850,000 people who have undiagnosed diabetes. The average prevalence of diabetes in the UK is 4.45% of the population, but there are variations between countries and regions. The proportion of people with diabetes increases with age. However, the incidence of diabetes is increasing in all age groups. Type 1 diabetes is increasing in children (especially those < 5 years of age), and type 2 diabetes is increasing, particularly in black and minority ethnic groups. In general, the prevalence of diabetes mellitus and its percentages in different populations is almost the same throughout the world.

Compounds **1−12**, **15−20**, **39−43** were tested for their antisweet activity. The antisweet activity was tested on a group of volunteers who held a solution of the tested compounds (5 mL of the specific compounds solution in 0.01 M NaHCO_3_) in their mouth for 3 min, spat, and rinsed with distilled water. The subjects were directed to taste 10 sucrose solutions from 0.1 to 1.0 M. The activity of the compound tested was expressed at the maximum concentration of a sucrose solution whose sweetness was suppressed completely. 0.5 mM of a solution of gymnemic acids I (**1**) and II (**2**) led a complete suppression of sweetness induced by 0.4 mM sucrose. More precisely, application of a 1 mM solution of **1** and **2** orally led to a complete suppression of sweetness induced by 0.2 and 0.4 M sucrose, respectively [30]. A 1.0 mM solution of gymnemic acids III (**3**), IV (**4**), V (**5**) and VI (**6**) led to a complete suppression of sweetness induced by 0.4 mM sucrose [31,32]. The difference between the structures of gymnemic acid III and gymnemic acid IV is only the absence or presence of a double bond in the acyl group. Kurihara *et al.* [33] have suggested that the acyl groups might play an important role in the generation of the antisweet activity. However, it seems that acyl groups only increase the antisweet activity, but are not essential [34]. A 0.5 mM solution of gymnemic acids VIII (**8**), IX (**9**) and X (**10**) leads to a complete suppression of the sweet taste of 0.2 M sucrose. The results are similar to those for gymnemic acids III and IV, which are acylated at C21 [35].

In general, it has been found that the antisweet activity of these saponins decreases with the decreasing number of acyl groups. In fact, a 0.5 mM solution of each of the gymnemic acids XI (**11**) and XII (**12**), as with gymnemic acids I and II, are able to suppress the perception of sweetness due to 0.4 M sucrose. The results suggest the antisweet activity of a triterpenes is directly proportional to the number of acyl groups present in the molecule [35]. A 0.5 M solution of gymnemic acids XV (**15**), XVI (**16**), XVII (**17**), and XVIII (**18**) completely suppressed the sweet taste of 0.4 M sucrose, showing the same activity of gymnemic acids I and II [36]. Gymnemic acid VII (**7**), prosapogenin (**20**) and gymnemagenin (**19**) were not active at all [30,31,32]. In particular, gymnemic acid IV is a multidirectional antihyperglycaemic agent with antisweet activity [31], glucose uptake inhibitory activity, and gut glycosidase inhibitory action [37]. Moreover, the blood glucose lowering effect of gymnemic acid IV was higher than glibenclamide, which was used as a control in streptozotocin-diabetic mice [29]. Most of these pharmacological effects may synergistically contribute to alleviating type 2 diabetes-related symptoms. Thus, gymnemic acid IV may be used as a prophylactic against diabetes through its different mechanisms of action [38].

A 1.0 mM solution of compounds **41**–**43** completely reduced the perceived sweetness of 0.1 M sucrose; these results correspond to half of the activity of gymnemic acids I−VI. Compounds **39** and **40** were not active [34].

The literature suggests that the pharmacological effects of *G. sylvestre* extract or its mixture of triterpenoids occur through mechanisms such as modulation of incretin activity, stimulation of insulin secretion and release, regeneration of β-endocrinocyte Langerhans islets, activation of enzymes responsible for glucose utilisation, reduction of glucose and fatty acid assimilation in the small intestine, and interference with the sensation of sweetness. It is known that hormones that regulate the formation and secretion of hormones by pancreas islets are activated in response to the entry of food into the intestine. Release of specific and well-known gastrointestinal hormones (GIP) into the portal vein in response to the intraduodenal administration of d-glucose in the presence of *G. sylvestre* extract enriched in gymnemic acids/triterpenoids by inhibitors of certain proposed glucose sensors and transporters in the intestinal lumen has been studied experimentally [39]. Intraduodenal administration of d-glucose caused a dose-dependent increase in the concentration of portal immunoreactive GIP. This suggests that the extract of *G. sylvestre* leaves or its constituents increases GIP secretion by endocrine k-cells in the small intestine [40]. The literature suggests that the hypoglycaemic activity of *G. sylvestre* is due to stimulating the release of insulin (and possibly the regeneration of Langerhans islet β-cells) and enzymes responsible for glucose utilisation and inhibition of glucose absorption in the bowel [27,41,42,43]. This means that the hypertrophy of β-endocrinocytes most likely occurs due to the effect of *G. sylvestre* on the increased secretion of GIP [44]. An autogenic hormone in the blood that was correlated with the hypoglycaemic effect was observed in experimental animals with an experimental form of immunodependent DM during a study of *G. sylvestre* activity.

In Russia, tests of mixtures consisting of *G. sylvestre* extracts of various purities (certified preparations of *G. sylvestre* dry extracts) in various concentrations in combination with extracts that intensify the antioxidant effect (grape stem) and possess immunomodulating and regenerative properties are underway. More than 30 different combinations have been investigated in detail *in vitro* and *in vivo*. Considering these facts, it is obvious that *G. sylvestre* is a source of biologically active substances.

The very broad spectrum of pharmacological activity for *G. sylvestre* indicates that the use of its extract or its components at various doses and in various combinations improves the condition of latent forms of DM (prediabetes), treats insulin-independent DM, prolongs the action of hypoglycaemic preparations, and regenerates β-cells for insulin-dependent and insulin-independent DM [45]. Despite the results of these studies, only a few metabolites of *G. sylvestre* have been tested for their effects on glucose uptake. Among them, gymnemosaponin V (**43**) and gymnemic acids I-IV increase the amount of insulin in blood plasma in mice with streptozotocin-induced DM after their administration [35,38]. The presence of these compounds, but perhaps not only them, could explain that, when *G. sylvestre* extract was used for 21 days after streptozotocin intoxication, it reliably reduced the levels of glucose and HbAlc in blood plasma, increased the insulin content, and normalised the concentration of high-density lipoproteins (HDL) [28]. The inhibitory activity of some triterpene glycosides was examined to determine their impact on the increase in serum glucose level in oral glucose-loaded rats. Gymnemoside-b (**33**) and gymnemic acids III, V, and VII were found to exhibit slight inhibitory activity towards the increase of glucose absorption after a single administration of 100 mg/kg, but gymnemic acid I and gymnemasaponin, lacked this activity at the same dose. Although the above compounds are included in one of three categories (acylated polyhydroxyoleanane 3-*O*-glucuronide) of glucose absorption inhibitors, their activity is much less than that of their analogues [46]. Gymnemic acids II and III showed potent inhibitory activities on glucose uptake, which were almost equivalent to those of oleanolic acid-3-*O*-glucuronide and Escin Ia. Gymnemoside-f (**37**), gymnemic acid IV and gymnemasapoin V were also found to inhibit glucose uptake, while gymenomosides-c (**34**), -d (**35**), and -e (**36**) lacked this activity. Gymnemic acids II and III showed no effect on the serum glucose levels in oral glucose-loaded rats. They exhibited potent inhibitory activity towards glucose uptake in rat small intestine fragments [37]. Gymnemic acid V is normally considered to inhibit glucose absorption in the small intestine at a concentration of 100 mg/kg. The inhibitory effect is particularly marked after 2 h of administration, with values that are quite comparable in absolute terms to those of elatosides, escins, and senegasaponins, which were used as controls. Gymnemic acid I shows an effect almost 50% higher than that of the control after 2 h despite appearing to be less active after 30 min and 1 h. A very similar effect that is only slightly smaller than the control or gymnemic acid V was demonstrated for gymnemic acid VII and gymnemosides-a (**32**) and -b (**33**). In order of decreasing activity up to a value of approximately 30% of the control were gymnemic acid II, IV, gymnemasaponins IV and V, gymnesaponin II (**40**) and gymnemic acid III. However, no apparent structure-activity relationship was observed [47].

In recent years interest in cancer prevention has grown steadily and urgently, therefore it would be particularly important to identify molecules that are able to prevent or avoid the processes of carcinogenesis due to substances of which substantially we can’t do without or can’t avoid in our daily life. Several anti-inflammatory substances are well known to inhibit the action of tumour promoters in the mouse skin carcinogenesis. The compound **46** was found to inhibit the inflammatory reaction induced by tumour promoters. This anti-inflammatory activity may play an important role in the mechanism of antitumor promotion as it has already been demonstrated [48,49]. The effects of compound **46** on the growth of HepG2 cells were measured. Its inhibitory effect was remarkably effective (ID_50_ was 25 µM) as it induced apoptosis at high dose, and with a dose-dependent manner [50,51]. Moreover, the application of antitumor-promoting triterpenoids is highly promising for protection against tumour formation, and many triterpenoids were tested *in vitro* and *in vivo* against the action of tumour promoter, 12-*O*-tetradecanoylphorbol 13-acetate (TPA) induced Epstein-Barr virus (EBV) activation in Raji cells. Recently, ursolic and oleanolic acids have been reported to be inhibitors of TPA and the dose responses of the acids were very similar to those of the antitumor promoters, such as retinoic and glycyrrhetinic acids and their analogues [51,52]. Thus some glycyrrhetinic acid-related compounds were found to be inhibitors of tumour promoter-induced phenomenon *in vitro*. Among these compounds, the compound **46** also proved to have *in vivo* antitumor-promoting activity in mouse skin tumour formation induced by 7,12-dimethylbenz[*a*]anthracene plus tumor promoters TPA [50]. Although its mechanism is unknown, the modulation of phospholipid metabolism appeared to be a very interesting aspect was proved that the inhibitory potency of the triterpenoids for the TPA-enhanced phospholipid synthesis is closely associated with their antitumor-promoting activity. Finally, a sulforhodamine B bioassay was used to determine the cytotoxicity of compound **44**. Its cytotoxic activity against four cultured human tumour cells was examined *in vitro*. The tumour cell lines were A549 (non small cell lung adenocarcinoma), SK-OV-3 (ovarian cancer cells), SK-MEL-2 (skin melanoma), and HCT15 (colon cancer cells) [53,54]. Doxorubicin was used as the positive control. The tested compound was essentially no cytotoxic [55]. A crude extract o mixture of compounds used in traditional medicine frequently contains components that have mutually opposing pharmacological activities beneficial for a specific disease. The higher potency of a crude drug is probably due to a synergistic effect among its component compounds, even though the activity of each compound is weak when used alone. This does not mean that it is not relevant to define a complete picture of the activities or biological properties of each compound. To identify a particular compound or a mixture of those with remarkable properties we should be able to deal with infusions or extracts that may have a different composition for an infinite number of reasons (location and/or harvesting season of the plant, particularly plant species, manner and time of extraction or partial purification *etc.*). Furthermore, the identification of one or of a few molecules suitable for the purpose would allow us to synthesize them, with a process as much as economically or timely convenient and to obtain pure products or well-defined composition.

**Table 1:** Common name and relative substituents of triterpenes with olean-12-ene skeleton.

No.	Common Name	R_1_	R_2_	R_3_	R_4_	R_5_	R_6_
**1**	Gymnemic acid I/3-*O*-*β*-d-Glucuronopyranosyl-21-*O*-tigloyl-28-*O*-acetyl gymnemagenin	GlcA	H	OH	OAc	OH	OTig
**2**	Gymnemic acid II/3-*O*-*β*-d-Glucuronopyranosyl-21-[*S*(+)-2-methyl-butyloyl]-28-*O*-acetyl gymnemagenin	GlcA	H	OH	OAc	OH	OMba
**3**	Gymnemic acid III/3-*O*-*β*-d-Glucuronopyranosyl-21-[*S*(+)-2-methyl-butyloyl]-gymnemagenin	GlcA	H	OH	OH	OH	OMba
**4**	Gymnemic acid IV/3-*O*-*β*-d-Glucuronopyranosyl-21-*O*-tigloyl-gymnemagenin	GlcA	H	OH	OH	OH	OTig
**5**	Gymnemic acid V/3-*O*-*β*-d-Glucuronopyranosyl-21,22-bis-tigloyl gymnemagenin	GlcA	H	OH	OH	OTig	OTig
**6**	Gymnemic acid VI/3-*O*-[*β*-d-Glucuronopyranosyl(1→3)-*β-*D-glucuronopyranosyl]-21-*O*-tigloyl gymnemagenin	A	H	OH	OH	OH	OTig
**7**	Gymnemic acid VII/3-*O*-*β*-d-Glucuronopyranosylgymnestrogenin	GlcA	H	OH	OH	H	OH
**8**	Gymnemic acid VIII	B	H	OH	OH	OH	OMba
**9**	Gymnemic acid IX	B	H	OH	OH	OH	OTig
**10**	Gymnemic acid X/3-*O*-*β*-d-Glucuronopyranosyl-28-*O*-acethyl gymnemagenin	GlcA	H	OH	OAc	OH	OH
**11**	Gymnemic acid XI/3-*O*-*β*-d-Glucuronopyranosyl-21,28-bis-*O*-tigloyl gymnemagenin	GlcA	H	OH	OTig	OH	OTig
**12**	Gymnemic acid XII/3-*O*-*β*-d-Glucuronopyranosyl (1→3)-*O*-*β*-d-glucopyranosyl-21-*O*-tigloyl-28-*O*-acetyl gymnemagenin	A	H	OH	OAc	OH	OTig
**13**	Gymnemic acid XIII	GlcA	H	OH	OMba	OH	OH
**14**	Gymnemic acid XIV	GlcA	H	OH	OTig	OH	OH
**15**	Gymnemic acid XV/3-*O*-*β*-d-Glucuronopiranosyl-21-*O*-2-methylbutyryl-22-*O*-2-methylcrotonoylgymnemagenin	GlcA	H	OH	OH	OTig	OMba
**16**	Gymnemic acid XVI/3-*O*-*β*-d-Glucuronopiranosyl 16,22-*O*-bis-2-methylcrotonoylgymnemagenin	GlcA	H	Tig	OH	OTig	OH
**17**	Gymnemic acid XVII/3-*O*-*β*-d-Glucuronopiranosyl-21-*O*-benzoyl gymnemagenin	GlcA	H	OH	OH	OH	OBz
**18**	Gymnemic acid XVIII/3-*O*-*β*-d-Glucuronopiranosyl-28-*O*-benzoyl gymnemagenin	GlcA	H	OH	OBz	OH	OH
**19**	Gymnemagenin/3*β*,16*β*,21*β*,22*α*,23,28-hexahydroxyolean-12-ene	H	H	OH	OH	OH	OH
**20**	Prosapogenin/3-*O*-*β*-d-Glucuronopyranosyl gymnemagenin	GlcA	H	OH	OH	OH	OH
**21**	12-Oleanene-3*β*,16*β*,23,28-tetrol/23-Hydroxylongispinogenin	H	H	OH	OH	H	H
**22**	3,16,23,28-*O*-Tetraacetyl 3*β*,16*β*,23,28-tetrahydroxyolean-12-ene	OAc	OAc	OAc	OAc	H	H
**23**	21-*O*-(2*S*)-Methylbutanoyl 3*β*,16*β*,21*β*,22*α*,23,28-hexahydroxyolean-12-ene	H	H	OH	OH	OH	OMba
**24**	28-*O*-acetyl 21-*O*-(2*S*)-methylbutanoyl 3*β*,16*β*,21*β*,22*α*,23,28-hexahydroxyolean-12-ene	H	H	OH	OAc	OH	OMba
**25**	3,16,22,23,28-*O*-Pentaacetyl 21-*O*-(2*S*)-methylbutanoyl 3*β*,16*β*,21*β*,22*α*,23,28-hexahydroxyolean-12-ene	OAc	OAc	OAc	OAc	OAc	OMba
**26**	21-*O*-Tigloyl 3*β*,16*β*,21*β*,22*α*,23,28-hexahydroxyolean-12-ene	H	H	OH	OH	OH	OTig
**27**	Gymnemanol/3*β*,16*β*,22*α*,23,28-pentahydroxyolean-12-en	H	H	OH	OH	OH	H
**28**	Gymnemasin A/3-*O*-[*β*-d-Gluconopyranosyl(1→3)-*β-*d-glucuronopyranosyl]-22-*O*-tigloyl gymnemanol	A	H	OH	OH	OTig	H
**29**	Gymnemasin B/3-*O*-[*β*-d-Gluconopyranosyl(1→3)-*β*-d-glucuronopyranosyl]-gymnemanol	A	H	OH	OH	OH	H
**30**	Gymnemasin C/3-*O*-*β*-d-glucuronopyranosyl-22-tigloyl gymnemanol	GlcA	H	OH	OH	OTig	H
**31**	Gymnemasin D/3-*O*-*β*-d-glucuronopyranosylgymnemanol	GlcA	H	OH	OH	OH	H
**32**	Gymnemoside-a/21-*O*-Tigloyl-22-*O*-acetylgymnemagenin 3-*O*-*β*-d-glucupyranosiduronic acid	GlcA	H	OH	OH	OAc	OTig
**33**	Gymnemoside-b/16-*O*-Acetyl-21-*O*-tigloyl-gymnemagenin 3-*O*-*β*-d-glucupyranosiduronic acid	GlcA	H	OAc	OH	OH	OTig
**34**	Gymnemoside-c/21-*O*-Benzoyl-28-*O*-acetylgymnemagenin 3-*O*-*β*-d-glucupyranosiduronic acid	GlcA	H	OH	OAc	OH	OBz
**35**	Gymnemoside-d/23-*O*-[*β*-d-Xylopyranosyl (1→6)-*β*-d-glucopyranosyl (1→6)-*β*-d-glucopyranosyl] gymnestrogenin	H	D	OH	OH	H	OH
**36**	Gymnemoside-e/23-*O*-[*β*-d-Xylopyranosyl(1→6)-*β*-d-glucopyranosyl (1→6)-*β*-d-glucopyranosyl]-28-*O*-[*β*-d-glucopyranosyl(1→6)-*β*-d-glucopyranosy] 23-hydroxylongispinogenin	H	D	OH	C	H	H
**37**	Gymnemoside-f/23-*O*-[*β*-d-Xylopyranosyl(1→6)-*β*-d-glucopyranosyl (1→6)-*β*-d-glucopyranosyl]-28-*O*-[9-d-glucopyranosyl (1→6)-β-d-glucopyarnosyl] 3*β*,16*β*,23,28-tetrahydroxyolean-18-ene	See Figure 2					
**38**	23-*O*-[*β*-d-Glucopyranosyl (1→6)-*β*-d-glucopyranosyl]-oleanene-3*β*,16*β*,23,28-tetrol/(+)-28-*O*-Desglucosylgymnemasaponin IV	H	C	OH	OH	H	H
**39**	Gymnemasaponin I	H	H	OH	Glc	H	H
**40**	Gymnemasaponin II	H	Glc	OH	Glc	H	H
**41**	Gymnemasaponin III	H	Glc	OH	C	H	H
**42**	Gymnemasaponin IV	H	C	OH	Glc	H	H
**43**	Gymnemasaponin V	H	C	OH	C	H	H
**44**	Gymnestrogenin/3*β*,16*β*,21*β*,23,28-Pentahydroxyolean-12-ene	H	H	OH	OH	H	OH
**45**	3*β*,16*α*,23,28-Tetrahydroxyolean-12-ene	See Figure 2					
**46**	3*β*,23,28-Trihydroxyolean-12-ene	H	H	H	OH	H	H
**47**	3*β*,16*β*,21*β*,23-Tetrahydroxyolean-12-ene	See Figure 2					
**48**	3*β*,16*β*,21*β*,23,28-Pentahydroxyolean-12-ene	H	H	OH	OH	H	OH
**49**	3*β*,16*β*,21*α*,23,28-Pentahydroxyolean-12-ene	See Figure 2					
**50**	3*β*,16*β*,23,28-Tetrahydroxyolean-13(18)-ene	See Figure 2					
**51**	16*β*,23,28-Trihydroxyolean-12-en-3-one	See Figure 2					
**52**	16*β*,21*β*,23,28-Tetrahydroxyolean-12-en-3-one	See Figure 2					
**53**	16*β*,21*β*,22*α*,23,28-Pentahydroxyolean-12-en-3-one						

For partial structures Glc, GlcA, A, B, C, and D see Figure 1.

**Figure 2:**
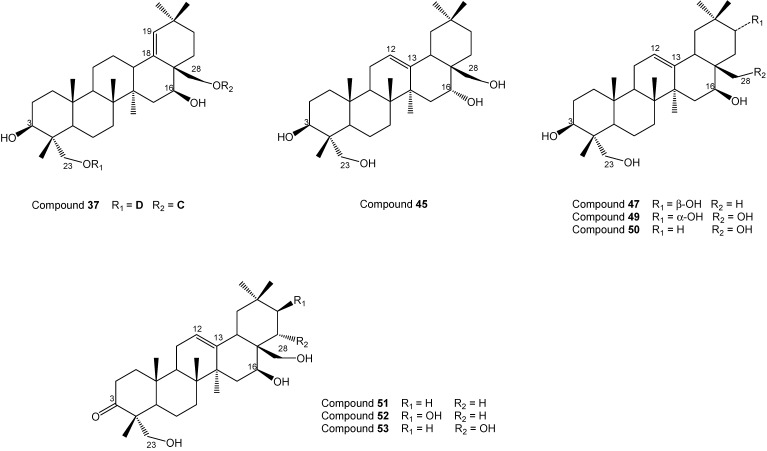
Chemical structures of compounds **37**, **45**, **47**, **49**–**53**

**Table 2:** Isolation and distribution of triterpenes in the different parts of the plants and their NMR spectral data.

No.	Part of the Plant	Extract	Ref.	Aspect	Solvents of NMR Spectra		
					^1^H-NMR	^13^C-NMR	Ref.
**1**	Leaves	H_2_O CH_3_OH	[31] [46]	/	C_5_D_5_N	C_5_D_5_N	[31,35]
**2**	Leaves	H_2_O CH_3_OH	[31] [46]	/	C_5_D_5_N	C_5_D_5_N	[31]
**3**	Leaves	H_2_O CH_3_OH	[31] [46]	Colourless powder	C_5_D_5_N	C_5_D_5_N	[30,31,56]
**4**	Leaves	H_2_O CH_3_OH	[31] [46]	Colourless powder	C_5_D_5_N	C_5_D_5_N	[30,31,56]
**5**	Leaves	H_2_O CH_3_OH	[32,46]	Colourless powder	C_5_D_5_N	C_5_D_5_N	[32,56]
**6**	Leaves	H_2_O	[32]	/	C_5_D_5_N	C_5_D_5_N	[32]
**7**	Leaves Leaves	H_2_O CH_3_OH	[32,46]	/	C_5_D_5_N	C_5_D_5_N	[32]
**8**	Leaves	H_2_O	[56]	Colourless powder	C_5_D_5_N+D_2_O	C_5_D_5_N+D_2_O	[56]
**9**	Leaves	H_2_O	[56]	Colourless powder	C_5_D_5_N+D_2_O	C_5_D_5_N+D_2_O	[56]
**10**	Leaves	H_2_O:EtOH (2:3)	[35]	Amorphous white powder	C_5_D_5_N	C_5_D_5_N	[35]
**11**	Leaves	H_2_O:EtOH (2:3)	[35]	Amorphous white powder	C_5_D_5_N	C_5_D_5_N	[35]
**12**	Leaves	H_2_O:EtOH (2:3)	[35]	Amorphous white powder	C_5_D_5_N	C_5_D_5_N	[35]
**13**	Leaves	H_2_O:EtOH (2:3)	[35]	Amorphous powder	C_5_D_5_N	C_5_D_5_N	[35]
**14**	Leaves	H_2_O:EtOH (2:3)	[35]	Amorphous powder	C_5_D_5_N	C_5_D_5_N	[35]
**15**	Leaves	H_2_O:EtOH (2:3)	[36]	Amorphous white powder	C_5_D_5_N	C_5_D_5_N	[36]
**16**	Leaves	H_2_O:EtOH (2:3)	[36]	Amorphous white powder	C_5_D_5_N	C_5_D_5_N	[36]
**17**	Leaves	H_2_O:EtOH (2:3)	[36]	Amorphous white powder	C_5_D_5_N	C_5_D_5_N	[36]
**18**	Leaves	H_2_O:EtOH (2:3)	[36]	Amorphous white powder	C_5_D_5_N	C_5_D_5_N	[36]
**19**	Leaves	Microwave	[51] [32]	/	C_5_D_5_N	C_5_D_5_N CDCl_3_+CD_3_OD	[31,56] [57]
	By synthesis						
**20**	By synthesis		[32]	/	C_5_D_5_N	C_5_D_5_N CD_3_OD	[31,56] [57]
**21**	Aerial parts	CH_2_Cl_2_	[58]	Amorphous powder	CD_3_OD	CD_3_OD	[58]
**22**	Aerial parts	CH_2_Cl_2_	[58]	Amorphous powder	CD_3_OD	CD_3_OD	[58]
**23**	Aerial parts	CH_2_Cl_2_	[58]	Amorphous powder	CD_3_OD	CD_3_OD	[58]
**24**	Aerial parts	CH_2_Cl_2_	[58]	Amorphous powder	CD_3_OD	CD_3_OD	[58]
**25**	Aerial parts	CH_2_Cl_2_	[58]	Amorphous powder	CD_3_OD	CD_3_OD	[58]
**26**	Aerial parts	CH_2_Cl_2_	[58]	Amorphous powder	CD_3_OD	CD_3_OD	[58]
**27**	By synthesis	[59]	Micro-needles	C_5_D_5_N	C_5_D_5_N	[59]	
**28**	Leaves	H_2_O:EtOH (1:1)	[59]	Amorphous powder	C_5_D_5_N	C_5_D_5_N	[59]
**29**	Leaves	H_2_O:EtOH (1:1)	[59]	Amorphous powder	C_5_D_5_N	C_5_D_5_N	[59]
**30**	Leaves	H_2_O:EtOH (1:1)	[59]	Amorphous powder	C_5_D_5_N	C_5_D_5_N	[59]
**31**	Leaves	H_2_O:EtOH (1:1)	[59]	Amorphous powder	C_5_D_5_N	C_5_D_5_N	[59]
**32**	Leaves	CH_3_OH	[46]	Colourless fine crystals	C_5_D_5_N	C_5_D_5_N	[46]
**33**	Leaves	CH_3_OH	[46]	Colourless fine crystals	C_5_D_5_N	C_5_D_5_N	[46]
**34**	Leaves	CH_3_OH	[46]	Colourless fine crystals	C_5_D_5_N	C_5_D_5_N	[37]
**35**	Leaves	CH_3_OH	[46]	Colourless fine crystals	C_5_D_5_N	C_5_D_5_N	[37]
**36**	Leaves	CH_3_OH	[46]	Colourless fine crystals	C_5_D_5_N	C_5_D_5_N	[37]
**37**	Leaves	CH_3_OH	[46]	Colourless fine crystals	C_5_D_5_N	C_5_D_5_N	[37]
**38**	By synthesis	[34]	/	C_5_D_5_N	C_5_D_5_N	[34]	
**39**	Leaves	H_2_O:EtOH (1:1)	[34]	/	C_5_D_5_N	C_5_D_5_N	[34]
**40**	Leaves	CH_3_OH H_2_O:EtOH (1:1)	[46] [34]	/	C_5_D_5_N	C_5_D_5_N	[34]
**41**	Leaves	H_2_O:EtOH (1:1)	[34]	/	C_5_D_5_N	C_5_D_5_N	[34]
**42**	Leaves	CH_3_OH H_2_O:EtOH (1:1)	[46] [34]	/	C_5_D_5_N	C_5_D_5_N	[34]
**43**	Leaves	CH_3_OH H_2_O:EtOH (1:1)	[46] [34]	/	C_5_D_5_N	C_5_D_5_N	[34]
**44**	Aerial parts	H_2_O	[32,60]	/	C_5_D_5_N	C_5_D_5_N	[32]
	By synthesis						
**45**	Aerial parts	H_2_O	[57]	Amorphous powder	CD_3_OD CDCl_3_	CD_3_OD CDCl_3_	[58] [61]
**46**	Aerial parts	CH_2_Cl_2_	[60]	/	CDCl_3_	CDCl_3_	[62]
**47**	Aerial parts	CH_2_Cl_2_	[60]	Amorphous powder	CD_3_OD	CD_3_OD	[60]
**48**	Aerial parts	CH_2_Cl_2_	[60]	/	C_5_D_5_N	C_5_D_5_N	[32]
**49**	Aerial parts	CH_2_Cl_2_	[60]	Amorphous powder	CD_3_OD	CD_3_OD	[60]
**50**	Aerial parts	CH_2_Cl_2_	[60]	Amorphous powder	CD_3_OD	CD_3_OD	[60]
**51**	Aerial parts	CH_2_Cl_2_	[60]	Amorphous powder	CD_3_OD	CD_3_OD	[60]
**52**	Aerial parts	CH_2_Cl_2_	[60]	Amorphous powder	CD_3_OD	CD_3_OD	[60]
**53**	Aerial parts	CH_2_Cl_2_	[60]	Amorphous powder	CD_3_OD	CD_3_OD	[60]

**Table 3:** Systematic names and physico-chemical characterization of triterpenes-1.

No.		Systematic Name		CAS		Molecular Formula		Molecular Weight		Melting Point °C		Ref.	
**1**		*β*-d-Glucopyranosiduronic acid, (3*β*,4*α*,16*β*,21*β*,22*α*)-28-(acetyloxy)-16,22,23-trihydroxy-21-[[(2*E*)-2-methyl-1-oxo-2-buten-1-yl]oxy]olean-12-en-3-yl		122,168-40-5		C_43_H_66_O_14_		806.97		211–212		[31]	
**2**		*β*-d-Glucopyranosiduronic acid, (3*β*,4*α*,16*β*,21*β*,22*α*)-28-(acetyloxy)-16,22,23-trihydroxy-21-[(2*S*)-2-methyl-1-oxobutoxy]olean-12-en-3-yl		122,144-48-3		C_43_H_68_O_14_		808.99		212–213		[31]	
**3**		*β*-d-Glucopyranosiduronic acid, (3*β*,4*α*,16*β*,21*β*,22*α*)-16,22,23,28-tetrahydroxy-21-[(2*S*)-2-methyl-1-oxobutoxy]olean-12-en-3-yl		122,074-65-1		C_41_H_66_O_13_		766.95		219–221 218–219		[56] [31]	
**4**		*β*-d-Glucopyranosiduronic acid, (3*β*,4*α*,16*β*,21*β*,22*α*)-16,22,23,28-tetrahydroxy-21-[[(2*E*)-2-methyl-1-oxo-2-buten-1-yl]oxy]olean-12-en-3-yl		121,903-96-6		C_41_H_64_O_13_		764.94		229–231 210–221		[56] [31]	
**5**		*β*-d-Glucopyranosiduronic acid, (3*β*,4*α*,16*β*,21*β*,22*α*)-16,23,28-trihydroxy-21,22-bis[[(2*E*)-2-methyl-1-oxo-2-butenyl]-oxy]olean-12-en-3-yl		121,903-99-9		C_46_H_70_O_14_		847.04		214–216 202–203		[56] [32]	
**6**		*β*-d-Glucopyranosiduronic acid, (3*β*,4*α*,16*β*,21*β*,22*α*)-16,22,23,28-tetrahydroxy-21-[[(2*E*)-2-methyl-1-oxo-2-butenyl]oxy]olean-12-en-3-yl 3-*O*-*β*-d-glucopyranosyl		121,903-98-8		C_47_H_74_O_18_		927.08		225–226		[32]	
**7**		*β*-d-Glucopyranosiduronic acid, (3*β*,4*α*,16*β*,21*β*)-16,21,23,28-tetrahydroxyolean-12-en-3-yl		121,903-97-7		C_36_H_58_O_11_		666.84		222–223		[32]	
**8**		*β*-d-Glucopyranosiduronic acid, [3*β*,4*α*,16*β*,21*β*(*S*),22*α*]-16,22,23,28-tetrahydroxy-21-(2-methyl-1-oxobutoxy)olean-12-en-3-yl 3-O-*β*-d-arabino-hexopyranos-2-ulos-1-yl		131,653-19-5		C_47_H_74_O_18_		927.08		218–220		[56]	
**9**		*β*-d-Glucopyranosiduronic acid, [3*β*,4*α*,16*β*,21*β*(*E*),22*α*]-16,22,23,28-tetrahydroxy-21-[(2-methyl-1-oxo-2-butenyl)oxy]-olean-12-en-3-yl 3-O-*β*-d-arabino-hexopyranos-2-ulos-1-yl		131,653-20-8		C_47_H_72_O_18_		925.06		222–224		[56]	
**10**		*β*-d-Glucopyranosiduronic acid, (3*β*,4*α*,16*β*,21*β*,22*α*)-28-(acetyloxy)-16,21,22,23-tetrahydroxyolean-12-en-3-yl		147,934-05-2		C_38_H_60_O_13_		724.86		210–212		[35]	
**11**		*β*-d-Glucopyranosiduronic acid, (3*β*,4*α*,16*β*,21*β*,22*α*)-16,22,23-trihydroxy-21,28-bis[[(2E)-2-methyl-1-oxo-2-butenyl]oxy]-olean-12-en-3-yl		147,899-35-2		C_46_H_70_O_14_		847.04		190–192		[35]	
**12**		*β*-d-Glucopyranosiduronic acid, (3*β*,4*α*,16*β*,21*β*,22*α*)-28-(acetyloxy)-16,22,23-trihydroxy-21-[[(2E)-2-methyl-1-oxo-2-butenyl]oxy]olean-12-en-3-yl 3-O-*β*-d-glucopyranosyl		147,899-36-3		C_49_H_76_O_19_		968.50		209–211		[35]	
**13**		*β*-d-Glucopyranosiduronic acid, (3*β*,4*α*,16*β*,21*β*,22*α*)-16,21,22,23-tetrahydroxy-28-[(2*S*)-2-methyl-1-oxobutoxy]olean-12-en-3-yl		155,023-61-3		C_41_H_66_O_13_		766.95		185–187		[35]	
**14**		*β*-d-Glucopyranosiduronic acid, (3*β*,4*α*,16*β*,21*β*,22*α*)-16,21,22,23-tetrahydroxy-28-[[(2*E*)-2-methyl-1-oxo-2-butenyl]oxy]olean-12-en-3-yl		155,023-62-4		C_41_H_64_O_13_		764.94		194–196		[35]	
**15**		*β*-d-Glucopyranosiduronic acid, (3*β*,4*α*,16*β*,21*β*,22*α*)-16,23,28-trihydroxy-22-[[(2*E*)-2-methyl-1-oxo-2-butenyl]oxy]-21-(2-methyl-1-oxobutoxy)olean-12-en-3-yl		154,977-74-9		C_46_H_72_O_14_		849.06		/		/	
**16**		*β*-d-Glucopyranosiduronic acid, (3*β*,4*α*,16*β*,21*β*,22*α*)-21,23,28-trihydroxy-16,22-bis[[(2*E*)-2-methyl-1-oxo-2-butenyl]oxy]olean-12-en-3-yl		154,977-75-0		C_46_H_70_O_14_		847.04		203–205		[36]	
**17**		Gymnemic acid XVII/*β*-d-Glucopyranosiduronic acid, (3*β*,4*α*,16*β*,21*β*,22*α*)-21-(benzoyloxy)-16,22,23,28-tetrahydroxyolean-12-en-3-yl		154,977-76-1		C_43_H_62_O_13_		786.94		211–213		[36]	
**18**		*β*-d-Glucopyranosiduronic acid, (3*β*,4*α*,16*β*,21*β*,22*α*)-28-(benzoyloxy)-16,21,22,23-tetrahydroxyolean-12-en-3-yl		154,977-77-2		C_43_H_62_O_13_		786.94		201–203		[36]	
**19**		Olean-12-ene-3,16,21,22,23,28-hexol, (3*β*,4*α*,16*β*,21*β*,22*α*)		22,467-07-8		C_30_H_50_O_6_		506.71		313–314 >300 328–335		[32,35] [56] [63]	
**20**		*β*-d-Glucopyranosiduronic acid, (3*β*,4*α*,16*β*,21*β*,22*α*)-3,16,21,22,23,28-hexahydroxyolean-12-en-3-yl		50,647-08-0		C_36_H_58_O_11_		666.84		230–231		[32,35]	
**21**		3*β*,16*β*,23,28-Tetrahydroxyolean-12-ene		42,483-24-9		C_30_H_50_O_4_		474.72		/		[58]	
**22**		3,16,23,28-O-Tetraacetyl 3β,16β,23,28-tetrahydroxyolean-12-ene		/		C_38_H_59_O_7_		627.87		/		[58]	
**23**		21-*O*-(2*S*)-Methylbutanoyl 3*β*,16*β*,21*β*,22*α*,23,28-hexahydroxyolean-12-ene		/		C_35_H_59_O_7_		591.84		/		[58]	
**24**		28-*O*-Acetyl 21-*O*-(2*S*)-methylbutanoyl 3*β*,16*β*,21*β*,22*α*,23,28-hexahydroxyolean-12-ene		/		C_37_H_61_O_7_		617.88		/		[58]	
**25**		3,16,22,23,28-*O*-Pentaacetyl 21-*O*-(2*S*)-methylbutanoyl 3*β*,16*β*,21*β*,22*α*,23,28-hexahydroxyolean-12-ene		/		C_45_H_68_O_11_		785.01		/		[58]	
**26**		21-*O*-Tigloyl 3*β*,16*β*,21*β*,22*α*,23,28-hexahydroxyolean-12-ene		/		C_35_H_57_O_6_		573.82		/		[58]	
**27**		Olean-12-ene-3,16,22,23,28-pentol, (3*β*,4*α*,16*β*,22*α*)		174,324-52-8		C_30_H_50_O_5_		490.72		284–285		[59]	
**28**		*β*-d-Glucopyranosiduronic acid, (3*β*,4*α*,16*β*,22*α*)-16,23,28-trihydroxy-22-[[(2*E*)-2-methyl-1-oxo-2-butenyl]oxy]olean-12-en-3-yl 3-*O*-*β*-d-glucopyranosyl		174,324-49-3		C_47_H_74_O_17_		910.49		215–217		[59]	
**29**		*β*-d-Glucopyranosiduronic acid, (3*β*,4*α*,16*β*,22*α*)-16,22,23,28-tetrahydroxyolean-12-en-3-yl 3-*O*-*β*-d-glucopyranosyl		174,324-48-2		C_42_H_68_O_16_		828.45		221–222		[59]	
**30**		*β*-d-Glucopyranosiduronic acid, (3*β*,4*α*,16*β*,22*α*)-16,23,28-trihydroxy-22-[[(2*E*)-2-methyl-1-oxo-2-butenyl]oxy]olean-12-en-3-yl		174,324-50-6		C_41_H_64_O_12_		748.44		212–214		[59]	
**31**		*β*-d-Glucopyranosiduronic acid, (3*β*,4*α*,16*β*,22*α*)-16,22,23,28-tetrahydroxyolean-12-en-3-yl		174,324-51-7		C_36_H_58_O_11_		666.40		220–221		[59]	
**32**		*β*-d-Glucopyranosiduronic acid, (3*β*,4*α*,16*β*,21*β*,22*α*)-22-(acetyloxy)-16,23,28-trihydroxy-21-[[(2*E*)-2-methyl-1-oxo-2-buten-1-yl]oxy]olean-12-en-3-yl		175,033-15-5		C_43_H_66_O_14_		806.98		207.0–208.5		[46]	
**33**		*β*-d-Glucopyranosiduronic acid, (3*β*,4*α*,16*β*,21*β*,22*α*)-16-(acetyloxy)-22,23,28-trihydroxy-21-[[(2*E*)-2-methyl-1-oxo-2-buten-1-yl]oxy]olean-12-en-3-yl		174,232-51-0		C_43_H_66_O_14_		806.98		211.5–213.0		[46]	
**34**		*β*-d-Glucopyranosiduronic acid, (3*β*,4*α*,16*β*,21*β*,22*α*)-28-(acetyloxy)-21-(benzoyloxy)-16,22,23-trihydroxyolean-12-en-3-yl		199,618-65-0		C_45_H_64_O_14_		828.98		211.5–213.0		[37]	
**35**		*β*-d-Glucopyranoside, (3*β*,4*α*,16*β*,21*β*)-3,16,21,28-tetrahydroxyolean-12-en-23-yl *O*-*β*-d-xylopyranosyl-(1→6)-*O*-*β*-d-glucopyranosyl-(1→6)-*β*-d-glucopyranosyl		199,618-66-1		C_47_H_78_O_19_		947.11		219.1–221.0		[37]	
**36**		*β*-d-Glucopyranoside, (3*β*,4*α*,16*β*)-3,16-dihydroxy-23-[(*O*-*β*-d-xylopyranosyl-(1→6)-*O*-*β*-d-glucopyranosyl-(1→6)-*β*-d-glucopyranosyl)oxy]olean-12-en-28-yl 6-*O*-*β*-d-glucopyranosyl		199,618-67-2		C_59_H_98_O_28_		1255.39		202.8–204.1		[37]	
**37**		*β*-d-Glucopyranoside, (3*β*,4*α*,16*β*)-3,16-dihydroxy-23-[(*O*-*β*-d-xylopyranosyl-(1→6)-*O*-*β*-d-glucopyranosyl-(1→6)-*β*-d-glucopyranosyl)oxy]olean-18-en-28-yl 6-*O*-*β*-d-glucopyranosyl		199,618-68-3		C_59_H_98_O_28_		1255.39		201.3–203.2		[37]	
**38**		*β*-d-Glucopyranoside, (3*β*,4*α*,16*β*)-3,16,28-trihydroxyolean-12-en-23-yl 6-*O*-*β*-d-glucopyranosyl		133,629-85-3		C_42_H_70_O_14_		799.00		173–175		[34]	
**39**		*β*-d-Glucopyranoside,(3*β*,4*α*,16*β*)-3,16,23-trihydroxyolean-12-en-28-yl		133,629-80-8		C_36_H_60_O_9_		636.86		184–185		[34]	
**40**		*β*-d-Glucopyranoside, (3*β*,4*α*,16*β*)-3,16-dihydroxyolean-12-ene-23,28-diyl bis		133,629-81-9		C_42_H_70_O_14_		799.00		190–192		[34]	
**41**		*β*-d-Glucopyranoside, (3*β*,4*α*,16*β*)-23-(*β*-d-glucopyranosyloxy)-3,16-dihydroxyolean-12-en-28-yl 6-*O*-*β*-d-glucopyranosyl		133,629-82-0		C_48_H_80_O_19_		961.14		203–205		[34]	
**42**		*β*-d-Glucopyranoside, (3*β*,4*α*,16*β*)-28-(*β*-d-glucopyranosyloxy)-3,16-dihydroxyolean-12-en-23-yl 6-*O*-*β*-d-glucopyranosyl		133,629-83-1		C_48_H_80_O_19_		961.14		201–203		[34]	
**43**		*β*-d-Glucopyranoside, (3*β*,4*α*,16*β*)-3,16-dihydroxyolean-12-ene-23,28-diyl bis[6-*O-β*-d-glucopyranosyl]		133,629-84-2		C_54_H_90_O_24_		1123.28		186–188		[34,37]	
**44**		Olean-12-ene-3,16,21,23,28-pentol, (3*β*,4*α*,16*β*,21*β*)		19,942-02-0		C_30_H_50_O_5_		490.72		290–291		[32]	
**45**		Olean-12-ene-3,16,23,28-tetrol, (3*β*,4*α*,16*β*)		23,887-98-1		C_30_H_50_O_4_		474.72				[64]	
**46**		(3*β*-Olean-12-ene-3,23,28-triol		35,043-82-4		C_30_H_50_O_3_		458.72		/		[62]	
**47**		(3*β*,16*β,*21*β-*Olean-12-ene-3,16,21,23-tetrol		1,447,214-81-4		C_30_H_50_O_4_		474.72		/		[62]	
**48**		(3*β*,16*β,*21*β-*Olean-12-ene-3,16,21,23,28-pentol		42,483-24-9		C_30_H_50_O_4_		474.72		/		[62]	
**49**		(3*β*,16*β*,21α-Olean-12-ene-3,16,21,23,28-pentol		1,447,214-84-7		C_30_H_50_O_5_		490.72		/		[62]	
**50**		(3*β*,16*β*-Olean-13(18)-ene-3,16,23,28-tetrol		26,540-63-6		C_30_H_50_O_4_		474.72		/		[62]	
**51**		16*β*,23,28-Tetrahydroxyolean-12-en-3-one		1,447,214-87-0		C_30_H_48_O_4_		472.70		/		[62]	
**52**		16*β*,21*β*,23,28-Tetrahydroxyolean-12-en-3-one		1,447,214-89-2		C_30_H_48_O_5_		488.70		/		[62]	
**53**		16 *β*,22*α*,23,28-Tetrahydroxyolean-12-en-3-one		1,447,214-91-6		C_30_H_48_O_5_		488.70		/		[62]	

**Table 4:** Physico-chemical characterization of triterpenes-2.

No.	MS Analysis	IR υ_max_, cm^−l^	[α]_D_ (c, MeOH)	Ref.
**1**	/	/	+36.7° (2.4)	[31]
**2**	/	/	+36.3° (1.5)	[31]
**3**	/	3400 (OH), 1715 (C=O)	+9.6° (0.39)	[56]
**4**	/	3400 (OH), 1700 (C=O)	+7.4° (0.21)	[56]
**5**	/ FAB(+): 892 [M + 2Na]^+^	3400 (OH), 1700 (C=O) /	+3.3° (0.30) +2.2° (3.6)	[56] [32]
**6**	FAB(+): 972 [M + 2Na]^+^	/	+11.7° (1.1)	[32]
**7**	FAB(+): 712 [M + 2Na]^+^	/	+9.6° (5.7)	[32]
**8**	HR-FAB(+): 949.4818 [M + Na]^+^	3450 (OH), 1730 (C=O)	+17.3° (0.74)	[56]
**9**	HR-FAB(+): 947.4681 [M + Na]^+^	3400 (OH), 1730 (C=O), 1700 (C=O)	+11.4° (0.70)	[56]
**10**	FAB(−): 723[M − H]^−^	3400 (OH), 1740 (C=O), 1610 (C=C), 1040 (OH)	+14.9° (2.3)	[35]
**11**	FAB(−): 845 [M − H]^−^	3400 (OH), 1740 (C=O), 1610 (C=C), 1040 (OH)	+1.7° (5.3)	[35]
**12**	FAB(−): 967 [M − H]^−^	3400 (OH), 1740 (C=O), 1720 (C=O), 1610 (C=C), 1040 (OH)	+11.7° (3.6)	[35]
**13**	FAB(−): 765 [M − H]^−^	3400 (OH), 1720 (C=O), 1600 (C=C), 1040 (OH)	+21.5° (3.5)	[35,36]
**14**	FAB(−): 763 [M − H]^−^	3380 (OH), 1705 (C=O), 1605 (C=C), 1060 (OH).	+7.6° (1.8)	[35,36]
**15**	FAB(−): 847 [M − H]^−^, 747 [M − H − C_5_H_8_O_2_]^−^, 745 [M − H − C_5_H_10_O_2_]^−^, 645 [M − H − C_5_H_8_O_2_-C_5_H_10_O_2_]^−^	3400 (OH), 1740 (C=O), 1720 (C=O), 1610 (C=C), 1040 (OH)	+7.2° (1.52)	[36]
**16**	FAB(−): 845 [M − H]^−^, 745 [M − H − C_5_H_8_O_2_]^−^, 645 [M – H − 2C_5_H_8_O_2_]^−^	3380 (OH), 1740 (C=O), 1650 (C=C), 1050 (OH)	−6.8° (2.96)	[36]
**17**	FAB(−): 785 [M − H]^−^, 663 [M − H − C_7_H_6_O_2_]^−^	3450 (OH), 1700 (C=O), 1720 (C=O), 1605(C=C), 1060 (OH)	+7.1° (2.96)	[36]
**18**	FAB(−): 785 [M − H]^−^, 663 [M − H − C_7_H_6_O_2_]^−^	3400 (OH), 1700 (C=O), 1650 (C=C), 1040 (OH)	+6.4° (1.71)	[36]
**19**	FAB(+): 529 [M + Na]^+^ FAB(+): 506 [M]^+^, 488 [M − H_2_O]^+^ HR-ESI-MS: 507.3678 [M + H]^+^	/ / 3328, 1111, 1089, 1037	+53.5° (1.8) +53.9° (0.75) −1.2° (0.19)	[32,35] [56] [58]
**20**	FAB(+): 705 [M + Na]^+^ 728 [M + 2Na]^+^	/ /	+8.4° (1.8) +8.4° (1.8)	[35] [32]
**21**	ESI-MS: 475.2 [M + H]^+^. HR-ESI-MS: 475.3780 [M + H]^+^ FAB(+): 497 [M + Na]^+^	3345, 1132, 1077, 1038	−0.67° (0.22) +32.0° (2.8)	[58] [34,61]
**22**	HR-ESI-MS: 643.4204 [M + H]^+^	3333, 1758, 1754, 1117, 1091, 1033	+50.0° (0.23)	[58]
**23**	HR-ESI-MS: 591.4254 [M + H]^+^	3370, 1747, 1118, 1096, 1046	+3.5° (0.21)	[58]
**24**	HR-ESI-MS: 633.4360 [M + H]^+^	3352, 1746, 1113, 1091, 1041	+16.5° (0.2 )	[58]
**25**	HR-ESI-MS: 801.4782 [M + H]^+^	3355, 1764, 1750, 1113, 1090, 1042	+2.5° (0.21)	[58]
**26**	HR-ESI-MS: 589.4099 [M + H]^+^	3352, 17,252, 1113, 1093, 1041	+3.5° (0.22)	[58]
**27**	EI: 490 [M]^+^, 472 [M − H_2_O]^+^, 454 [M − 2H_2_O]^+^ 441 [M − H_2_O − CH_2_OH]^+^, 436 [M − 3H_2_O]^+^	3350 (OH)	+51.5° (1.0)	[59]
**28**	FAB(+): 933 [M + Na]^+^. FAB(−): 909 [M − H]^−^	3400 (OH), 1715 (C=O), 1600 (C=C)	+15° (1.5)	[59]
**29**	FAB(−): 827 [M − H]^−^	3420 (OH), 1710 (C=O)	+18.5° (1.0)	[59]
**30**	FAB(−): 747 [M − H]^−^	3410 (OH), 1715 (C=O)	+12.5° (1.0)	[59]
**31**	FAB(−): 665 [M − H]^−^	3425 (OH),1715 (C=O)	+8° (0.9)	[59]
**32**	HR-FAB(−): 805.4385 [M − H]^−^ HR-FAB(+): 829.4430 [M − Na]^+^	3453, 1721, 1649, 1040	+4.7° (0.1)	[46]
**33**	HR-FAB(−): 805.4404 [M − H]^−^ HR-FAB(+): 829.4428 [M − Na]^+^	3445, 1718, 1649, 1044	+6.6° (0.1)	[46]
**34**	HR-FAB(+): 829.4360 [M + Na]^+^ FAB(+): 851 [M + Na]^+^. FAB(−): 827 [M − H]^−^	3445, 1718, 1649, 1044	+6.6° (0.1)	[37]
**35**	HR-FAB(+): 969.5050 [M + Na]^+^ FAB(+): 991 [M + 2Na − H]^+^, 969 [M + Na]^+^. FAB(−): 945 [M − H]^−^	3410, 1044	+13.4° (0.1)	[37]
**36**	HR-FAB(−): 1253.6154 [M − H]−, FAB(+): 1277 [M + Na]^+^. FAB(−): 1253 [M − H]^−^	3410, 1044	+14.8° (0.1)	[37]
**37**	HR-FAB(−): 1253.6167 [M − H]^−^ FAB(+): 1277 [M + Na]^+^. FAB(−): 1253 [M − H]^−^	3431, 1044	−8.9° (0.1)	[37]
**38**	FAB(+): 821 [M + Na]^+^	/	+12.1° (1.1)	[34,61]
**39**	FAB(+): 659 [M + Na]^+^	/	+9.3° (3.5)	[34,61]
**40**	FAB(+): 821 [M + Na]^+^	/	+1.9° (2.6)	[34,61]
**41**	FAB(+): 983 [M + Na]+	/	−11.6° (1.1)	[34,61]
**42**	FAB(+): 98 3[M + Na]+	/	−1.1° (1.9)	[34,61]
**43**	FAB(+): 1145 [M + Na]+	/	−6.2°(1.9)	[34,61]
**44**	FAB(+): 712 [M+2Na]^+^	/	+53.1° (2.4)	[32]
**45**	ESI-MS: 475.2 [M + H]^+^. HR-ESI-MS: 475.3780 [M + H]^+^	3333, 1758, 1754, 1117, 1091, 1033	−0.67° (0.22)	[36]
**46**	HR-FAB(+): 481.3720 [M + Na]^+^	3344, 2930, 2857, 1725, 1459, 756	/	[62]
**47**	ESI-MS: 475.2 [M + H]^+^. HR-ESI-MS: 475.3769 [M + H]^+^	3347, 1130, 1080, 1035	+20.5° (0.27)	[60]
**48**	/	/	+53.1° (2.4)	[32]
**49**	ESI-MS: 491.4 [M + H]^+^. HR-ESI-MS: 491.3728 [M + H]^+^	3330, 1115, 1095, 1035	+32.3° (0.23)	[60]
**50**	ESI-MS: 475.5 [M + H]^+^. HR-ESI-MS: 475.3745 [M + H]^+^	3334, 1112, 1087, 1034	−1.2° (0.19)	[60]
**51**	ESI-MS: 473.2 [M + H]^+^. HR-ESI-MS: 473.3620 [M + H]^+^	3376, 1722, 1118, 1090, 1045	+25.5° (0.21)	[60]
**52**	ESI-MS: 489.2 [M + H]^+^. HR-ESI-MS: 489.3563 [M + H]^+^	3350, 1724, 1118, 1090, 1045	+26.5° (0.22)	[60]
**53**	ESI-MS: 505.3 [M + H]^+^. HR-ESI-MS: 505.3500 [M + H]^+^	30, 1723, 1128, 1080, 1050	+23.1° (0.27)	[60]

**Table 5:** Biological properties associated with the triterpenes with a focus on their potential chemotherapeutic applications.

No.	Activity	Ref.
**1**	Antisweet activity Glucose uptake in rat small intestinal fragment Increase of serum glucose level in oral glucose-loaded rats Anti-hyperglycemic activity	[31,35,38] [37,38] [46,47] [38]
**2**	Antisweet activity Glucose uptake in rat small intestinal fragment Increase of serum glucose level in oral glucose-loaded rats Anti-hyperglycemic activity	[31,35,38] [37,38] [46,47] [38]
**3**	Antisweet activity Glucose uptake in rat small intestinal fragment Increase of serum glucose level in oral glucose-loaded rats Anti-hyperglycemic activity	[30,31,35,38] [37,38] [46,47] [38]
**4**	Antisweet activity Glucose uptake in rat small intestinal fragment Increase of serum glucose level in oral glucose-loaded rats Anti-hyperglycemic activity Gut glycosidase inhibition	[30,31,35,38] [37,38] [46,47] [38,65] [38]
**5**	Antisweet activity Increase of serum glucose level in oral glucose-loaded rats Anti-hyperglycemic activity Glucose uptake in rat small intestinal fragment	[32,38] [46,47,65] [38] [38]
**6**	Antisweet activity	[32]
**7**	Antisweet activity Increase of serum glucose level in oral glucose-loaded rats	[32] [46,47]
**10**	Antisweet activity	[35]
**11**	Antisweet activity	[35]
**12**	Antisweet activity	[35]
**13**	Antisweet activity	[35]
**14**	Antisweet activity	[35]
**15**	Antisweet activity	[36]
**16**	Antisweet activity	[36]
**17**	Antisweet activity	[36]
**18**	Antisweet activity	[36]
**19**	Antisweet activity	[31]
	Pharmacokinetic study: determination of gymnemagenin in rat plasma using HPLC-MS/MS	[66]
**20**	Antisweet activity	[31]
**27**	Inhibition of the 11β-hydroxysteroid dehydrogenase type 1	[63,66]
**28**	Hypoglycemic and antihyperglycemic effect in rats	[61]
**29**	Hypoglycemic and antihyperglycemic effect in rats	[61]
**30**	Hypoglycemic and antihyperglycemic effect in rats	[61]
**31**	Hypoglycemic and antihyperglycemic effect in rats	[61]
**32**	Increase of serum glucose level in oral glucose-loaded rats	[46,47]
**33**	Increase of serum glucose level in oral glucose-loaded rats	[46,47]
**34**	Glucose uptake in rat small intestinal fragment	[37]
**35**	Glucose uptake in rat small intestinal fragment	[37]
**36**	Glucose uptake in rat small intestinal fragment	[37]
**37**	Glucose uptake in rat small intestinal fragment	[37]
**39**	Antisweet activity	[34]
**40**	Antisweet activity Glucose uptake in rat small intestinal fragment	[34] [46,47]
**41**	Antisweet activity	[34]
**42**	Antisweet activity Glucose uptake in rat small intestinal fragment	[34] [46,47]
**43**	Antisweet activity Glucose uptake in rat small intestinal fragment	[34] [37,47,59]
**44**	Inhibitory effects on human tumor cell lines (A549, SK-OV-3, SK-MEL-2, and HCT15) *in vitro* using the sulforhodamine B (SRB) assay	[55]
**46**	*In vivo* antitumor-promoting activity in mouse skin tumor	[50]
	Inhibition of the tumor-promoting action of 12-*O*-tetradecanoylphorbol 13-acetate	[48,49]
	Inhibition of phospholipid synthesis by 12-*O*-tetradecanoylphorbol-13-acetate	[67]
	*In vitro* in human uterus cancer cells	
	Anti-inflammatory activity and also to inhibit liver carcinogenesis and tumor growth	[51]

## 3. Conclusions

The market of natural substances is very attractive for its economic impact, which, on the other hand grows continuously. Research into new natural substances that can be used in the pharmaceutical, agrochemical and agro-industrial production of drugs, biopesticides and food additives has grown in recent years. *Gymnema sylvestre* is a relevant specific example of a plant very interesting for its numerous pharmacological properties, which include antidiabetic, anticarcinogenic, and neuroprotective effects, used as a medicinal plant in Asia for thousands of years. Its properties are attributed to triterpenoidic saponins. In light of the considerable interest generated in the chemistry and pharmacological properties of *G. sylvestre* triterpenes and their analogues, this review summarises the available literature on these promising bioactive natural products. The review shows the results about the isolation, chemistry and bioactivity of the triterpenoids oxidised at C-23, which are schematically presented in few tables, in particular, their isolation, distribution in different parts of the plant, and their NMR spectral data; their names and physico-chemical characterisation; and the biological properties associated with these compounds, with a focus on their potential chemotherapeutic applications.
